# Simultaneous Two- and Three-Photon Deep Imaging of Autofluorescence in Bacterial Communities

**DOI:** 10.3390/s24020667

**Published:** 2024-01-20

**Authors:** Alma Fernández, Anton Classen, Nityakalyani Josyula, James T. Florence, Alexei V. Sokolov, Marlan O. Scully, Paul Straight, Aart J. Verhoef

**Affiliations:** 1 Department of Soil and Crop Sciences, Texas A&M University, TAMU 2474, College Station, TX 77843, USA; antonclassen89@gmail.com; 2 Institute for Quantum Science & Engineering, Texas A&M University, TAMU 4242, College Station, TX 77843, USA; sokol@tamu.edu (A.V.S.); scully@tamu.edu (M.O.S.); 3 Department of Biochemistry and Biophysics, Texas A&M University, TAMU 2128, College Station, TX 77843, USA; nityakalyani.josyula@ag.tamu.edu (N.J.); paul.straight@ag.tamu.edu (P.S.); 4 Department of Physics & Astronomy, Texas A&M University, TAMU 4242, College Station, TX 77843, USA; jtflorence@tamu.edu

**Keywords:** multiphoton microscopy, bacterial communities, deep imaging, three-photon excitation, autofluorescence

## Abstract

The intrinsic fluorescence of bacterial samples has a proven potential for label-free bacterial characterization, monitoring bacterial metabolic functions, and as a mechanism for tracking the transport of relevant components through vesicles. The reduced scattering and axial confinement of the excitation offered by multiphoton imaging can be used to overcome some of the limitations of single-photon excitation (e.g., scattering and out-of-plane photobleaching) to the imaging of bacterial communities. In this work, we demonstrate in vivo multi-photon microscopy imaging of Streptomyces bacterial communities, based on the excitation of blue endogenous fluorophores, using an ultrafast Yb-fiber laser amplifier. Its parameters, such as the pulse energy, duration, wavelength, and repetition rate, enable in vivo multicolor imaging with a single source through the simultaneous two- and three-photon excitation of different fluorophores. Three-photon excitation at 1040 nm allows fluorophores with blue and green emission spectra to be addressed (and their corresponding ultraviolet and blue single-photon excitation wavelengths, respectively), and two-photon excitation at the same wavelength allows fluorophores with yellow, orange, or red emission spectra to be addressed (and their corresponding green, yellow, and orange single-photon excitation wavelengths). We demonstrate that three-photon excitation allows imaging over a depth range of more than 6 effective attenuation lengths to take place, corresponding to an 800 micrometer depth of imaging, in samples with a high density of fluorescent structures.

## 1. Introduction

Live bacteria possess a variety of endogenous fluorescent biological compounds with specific intrinsic excitation and emission wavelengths that can be used in applications such as label-free probes for bacterial detection and characterization purposes, for monitoring bacterial metabolic functions [1], and as a tracking mechanism of outer-membrane carrier vesicles [2], to mention a few. One microbial community of interest is the *Streptomyces* bacterial community. *Streptomyces* are a genus of filamentous bacteria that develop into mycelial communities of intertwined filaments. They are of great biotechnological and pharmaceutical relevance, especially for their ability to produce numerous clinically useful natural products such as antibacterial and antitumor compounds [3]. While a major focus for *Streptomyces* research is the production of bioactive molecules, these organisms are also of great microbiological interest for their growth and development of spores.

Many studies have been carried out using confocal microscopy (with single-photon excitation, 1PE) to image *Streptomyces*, looking either at the entire mycelium or single filaments for various purposes [3,4,5,6]. The single-photon excitation wavelengths of many endogenous fluorophores in bacteria and other biological samples, such as amino acids, enzymes, and co-enzymes, lie in the 240 nm to 500 nm wavelength [1]. The single-photon excitation of fluorophores in the deep blue and ultraviolet (UV) spectral range is especially disadvantageous because of the strong scattering and absorption in living tissue, which limit the imaging penetration depth at these wavelengths. In addition, the associated increased phototoxicity of the shorter excitation wavelengths can compromise sample viability. Recent studies involving imaging sub-surface features in *Streptomyces* mycelia (depths of hundreds of micrometers) were performed by mechanically sectioning the mycelium (creating vertically oriented slices) before confocal imaging [7,8,9]. This methodology is destructive to the sample and requires a more involved process of sample preparation.

As an alternative to confocal microscopy (with 1PE), laser scanning multiphoton fluorescence microscopy, often combined with genetically encoded green and red fluorophores, has become a powerful tool for high-resolution imaging into high-scattering biological samples at depths beyond 1 mm [10,11]. As compared to two-photon (2P) fluorescence excitation, three-photon (3P) fluorescence excitation [12] has been particularly successful for achieving unprecedented deep-tissue imaging in high-scattering samples (like the mouse brain) due to the use of longer excitation wavelengths and higher nonlinear confinement. The use of a longer wavelength for 3P excitation reduces tissue scattering, which is advantageous for increasing imaging depth. In addition, the higher-order nonlinear process involved in 3P excitation allows for a greater suppression of out-of-focus excitation as compared to 2P excitation, which increases the signal-to-background ratios (SBRs). Despite the demonstrated capability of multiphoton laser scanning fluorescence microscopy (LSM) for the high-resolution in vivo deep imaging of optically dense biological tissue, its application to imaging bacterial communities has been very scarce, and to the best of our knowledge, it has been limited to 2P microscopy demonstrations [13,14,15,16,17,18,19,20], with no studies undertaken using 3P microscopy.

Most of the pioneering work in 3P microscopy has been applied to neuroimaging, in combination with the excitation of genetically encoded green and red fluorophores, using excitation sources operating around 1300 nm and 1700 nm, respectively. Recent demonstrations of 3P excitation with tunable Ti:sapphire oscillators, which are routinely used for 2P microscopy, point out the limitations in imaging speed and imaging depth due to insufficient excitation power and increased photodamage risk from the high average power needed with the lower efficiency of 3P excitation [21,22]. Nonetheless, shorter-wavelength excitation sources (e.g., in the 700–1100 nm spectral window) delivering high-energy femtosecond pulses at low MHz repetition rates are very attractive for applications targeting endogenous fluorescence in the UV and blue spectral region with 3P excitation. Three-photon microscopy imaging using an ultrafast 1030 nm Yb-fiber laser has been investigated for in vivo imaging in mouse intestine tissue; however, the imaging speed of the in-vivo study was not satisfactory due to the low repetition rate (~100 kHz) of the laser [22], and the longer pulse duration (~750 fs) of the Yb-fiber laser system used also reduces the signal generation efficiency (which inversely scales with the square of the pulse duration for 3P excited fluorescence).

**Figure 1:**
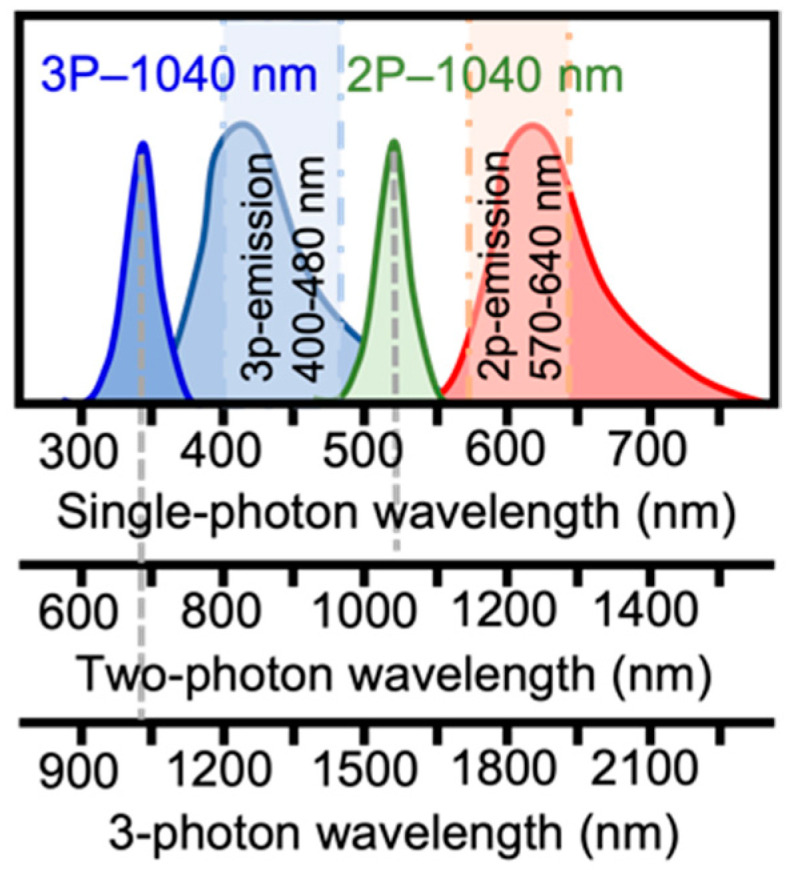
Example excitation spectra and emission spectra for fluorophores in a multicolor, multiphoton imaging experiment. On the x-axes, we show (from top to bottom) the wavelength corresponding to single-photon excitation and emission, 2-photon excitation, and 3-photon excitation, respectively.

In this paper, we investigate, for the first time (to our knowledge), the use of 3P-LSM to imaging *Streptomyces* bacterial communities. We demonstrate, for the first time, in vivo simultaneous deep 2P and 3P label-free imaging of endogenous fluorophores emitting in the red, green, and blue spectral regions in Streptomyces mycelia using a single excitation wavelength provided by a monolithic Yb-fiber laser (Figure 1). We show how the use of 3P LSM was pivotal to overcome strong out-of-plane photobleaching effects in *Streptomyces* samples when excited using deep-blue or UV light sources, while stable blue fluorescence without out-of-plane photobleaching was detected when using 3P excitation on the samples.

The good pulse compressibility of higher-order dispersion terms is challenging in such all-fiber systems [23,24,25], but it is necessary for efficient (nonlinear) 2P excitation [25,26,27] and even more critical for 3P excitation [28]. Our imaging system allows for simultaneous 2P and 3P imaging with single-wavelength excitation by employing appropriate bandpass filters in the detection path. The single excitation wavelength of 1040 nm, derived directly from the ultrafast Yb-fiber laser, eliminates the need for optical parametric amplification schemes, thus reducing the complexity of the equipment needed for 3P imaging. The signal strength and imaging contrast as a function of depth are characterized and compared for 2P and 3P microscopy. We show the advantages of this imaging system in generating stable fluorescence signals. Our experiments are supported by simulations of the 2P and 3P point-spread function (PSF) and SBR. Given the degree of scattering in biological samples, the results emphasize the advantages of having 3P imaging capabilities, especially for blue-shifted fluorophores that require 3P excitation. For a sample with a 120 µm scattering length (i.e., a medium-dense sample), by working at the maximum power currently available from our Yb-fiber amplifier, we could image up to a depth of 780 µm.

## 2. Materials and Methods

### 2.1. Bacterial Communities

**Figure 2:**
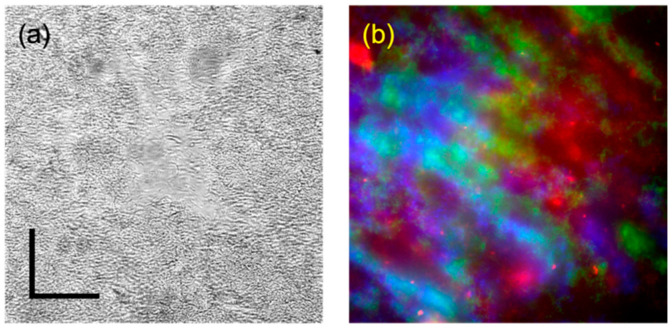
(**a**) Brightfield and (**b**) widefield epifluorescence images (composite of 405/440 (blue), 488/520 (green), and 560/605 (red) (excitation/emission filter central wavelength)) of a thin *S. aizunensis* sample (~3 µL of sample sandwiched between a microscope slide and cover slip), showing the filaments formed in the cultures. The strong scattering from this sample causes a significant blur, even when the focal plane is directly below the cover slip. Scale bars: 100 µm.

The bacterial strain used in this study is *Streptomyces aizunensis* NRRL B-11277. Freshly isolated spores of *S. aizunensis* (107 spores/mL) were inoculated in 25 mL of GYM7 media (0.4% *w*/*v* D-glucose, 0.4% yeast extract, 0.4% malt extract, 100 mM of MOPS, 2.5 mM of KH_2_PO_4_, and 2.5 mM of K_2_HPO_4_, pH 7.0). The cultures were grown at 30 °C and 225 rpm in a shaker incubator. At the end of 2 days (48 h post inoculation), the culture was centrifuged at 4000 rpm. The supernatant was discarded, and the pelleted cells were used to prepare samples, with varied thickness and naturally varying density, for imaging. Figure 2 shows brightfield images taken from typical samples. Thin (~30 µm) samples were prepared by applying a small (~3 µL) droplet of pelleted cells onto a microscope slide, subsequently covered with a cover slip. Thick samples were prepared by sticking a (1 mm or thicker) plastic ring onto a microscope slide, filling the resulting well with pelleted cells, and covering the filled well with a cover slip. Note that both the brightfield images and widefield epi-fluorescence images of these thick samples do not provide a useable contrast.

### 2.2. Multiphoton Multicolor Imaging

**Figure 3:**
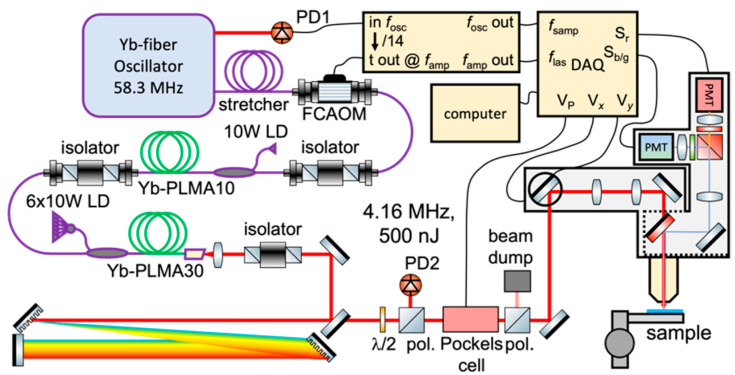
Schematic of our laser and microscope system. The repetition rate of the laser was controlled by choosing an appropriate divisor to the oscillator pulse train with the timing electronics. The detection electronics’ sampling rate was set with the oscillator pulse train, and then synchronized to the amplifier output pulses.

**Figure 4:**
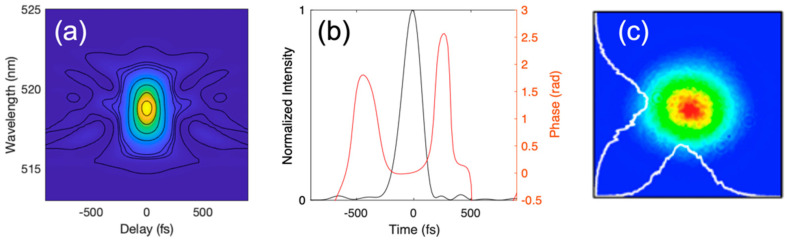
Characterization of the output beam from our femtosecond fiber laser. The pulses were characterized using second-harmonic frequency-resolved optical gating (**a**,**b**), showing a pulse duration of 180 fs with minimal satellite pulses. (**c**) The far-field beam profile, measured with a beam profiler camera. The profile is nearly perfectly Gaussian, owing to only the fundamental mode being launched in the large-mode area final amplifier stage.

A simplified schematic of the multimodal setup for 2P and 3P simultaneous imaging is shown in Figure 3. The excitation source is a homebuilt monolithic Yb-fiber laser source, consisting of an all-normal-dispersion single-mode fiber oscillator, a single-mode fiber pre-amplifier, and a 30-µm core Yb-doped large-mode-area fiber amplifier. It is important to note that all components in the amplifier were fusion-spliced together, and therefore, mechanical instabilities were fully eliminated. The fiber amplifier system design has been discussed in detail elsewhere [24,29]. Good pulse compressibility is achieved by ensuring sufficient stretching of the low energetic pulses in a dispersion-compensation fiber and single-mode fiber, arranged in such a way that nonlinearities due to the high confinement of the beam in the fiber cores are minimized and by choosing their lengths carefully to match the dispersion of the system to the compressor dispersion in order to compensate for higher-order dispersion terms. The system delivers 180 fs, high-quality pulses centered at 1040 nm, with an M2 of <1.1 (see Figure 4). Good pulse compressibility and beam quality benefit signal generation efficiency. The repetition rate can be varied between tens of kHz and ~20 MHz by varying the down-counting rate on the acousto-optic modulator (pulse picker). It is also possible to operate the laser at the master oscillator repetition rate, which, for our system, is 58 MHz. For this experiment, we worked with a repetition rate of 4.17 MHz, which can be considered to provide a good compromise between imaging speed and signal strength. At this repetition rate, we typically operate the laser with an output pulse energy of 400 nJ, which is achieved at roughly 35% of the available pump power of the LMA amplification stage. Several microjoules of energy can be achieved from this system (at lower repetition rates), but the pulse energy requirements for our imaging experiments were well below 400 nJ. Compared to currently available commercial systems that can deliver sub-200 fs pulses at 1040 nm, our system has a much lower power consumption, as no optical parametric amplification is needed because the 1040 nm excitation pulse directly from the fiber laser allows for simultaneous 2P and 3P excitation, and our laser does not require water cooling. Pulse compression at the sample plane was achieved by means of a high-throughput grating compressor (over 96% overall efficiency) that matched the overall spectral phase (dispersion) introduced by the fiber amplifier and also accounted for additional material dispersion introduced into the microscope beam’s path. The imaging power was controlled using a Pockels cell (M350-80LA, ConOptics, Danbury, CT, USA). A pair of galvanometric mirrors (MDR, Sutter Instrument, Novato, CA, USA) that were optically conjugated to the back focal plane of a 25×, 1.05 numerical aperture (NA) objective (XLPLN25XWMP2, Olympus, Tokyo, Japan) steered the excitation focus in the imaging plane. The objective was mounted on a 3-axis micromanipulator stage (MPC-200, Sutter Instrument) to position the objective over the sample, and a piezoelectric stage (nPFocus 400, nPoint, Middleton, WI, USA) to translate the focus axially with high precision. Image acquisition was carried out using ScanImage (Vidrio Technologies, Leesburg, VA, USA).

The applied laser pulse energy at the sample can be varied between 0 and ~60 nJ, corresponding to a power of ~250 mW. Adjusting the pulse energy on the sample is used to optimize the retrieved images when changing the imaging depth (from the sample surface to >500 µm below the surface), due to scattering in the sample and depending on fluorophore excitation efficiency. The extinction ratio that can be achieved with the Pockels cell is measured to be ~300-fold, which allows us to provide constant excitation energy within focus while changing the imaging depth over more than 5 scattering lengths. To allow attenuation of the excitation power by more than 300 times, an additional half-wave plate and polarizer in front of the Pockels cell were added. Note that the actual average power on the sample at the maximum pulse energy was, in fact, lower than ~250 mW, as the Pockels cell is programmed to reduce the laser power to zero during galvo flyback, while adjusting the objective z-position, etc. The back aperture of the objective was not overfilled, such that the effective excitation NA in our experiments was slightly lower than the nominal NA of the objective. The fluorescence signal was collected by the same objective. Two detection channels equipped with GaAsP PMTs (H10770PA-40SEL, Hamamatsu, Japan) were used to detect the fluorescence, in an arrangement that allowed for ultra-large field-of-view non-de-scanned detection. The signal was reflected by a long-pass dichroic beamsplitter (FF735-Di02-25x36, Semrock, Rochester, NY, USA). The signals were then separated into the two detection channels with a second long-pass dichroic (T565lpxr, Chroma, Irvine, CA, USA) and bandpass filters centered at 440 nm for 3P detection (‘blue’, ET440/80m-2p, Chroma), 605 nm for 2P detection (‘orange/red’, ET605/70m-2p, Chroma), and 525 nm for mixed 2P and 3P detection (‘green’, ET525/70m-2p, Chroma). The arrangement required us to exchange the ‘blue’ (440 nm) and ‘green’ (525 nm) filters between measurements to detect both ‘blue’ and ‘green’ fluorescence for a given sample. As the ‘orange/red’ fluorescence can be detected in parallel with both measurements, we use the two ‘orange/red’ images to confirm sample stability (motion, photo-bleaching, and damage) between measurements.

Our multiphoton microscopy images were acquired by scanning a focused laser beam over the sample. Images were formed by recording the signal at each laser position and assigning the measured value to the corresponding pixel. The signal quality depends on the time spent to acquire the signal for each pixel, and increasing this time increases the image quality. However, using slow beam scanning also tends to increase the probability to locally damage the sample. Scanning the image fast and repeating the image scan multiple times can reduce the risk of damaging the sample and improve the image quality. An additional advantage of this strategy is that hypothetical slow variations (drift) in laser parameters will be homogenized over all pixels rather than cause time-evolution artifacts on the image. Sample movement will appear as image distortions when scanning the beam slowly to acquire the image in one frame, or as image blur when scanning the beam quickly and averaging several (quick) frames. For our measurements, we scanned the laser beam as fast as our galvanometric mirrors allowed (i.e., the fast-scanning mirror swept the beam over a line in 1 ms), and averaged over a significant number of frames.

### 2.3. Image Analysis and Processing

We use custom MATLAB scripts and built-in functions from FIJI to analyze our images. To obtain the fluorescence intensity as a function of laser power, we saved the images acquired at different laser powers into a multicolor image stack, with increasing laser power with increasing slices. In the multicolor image stack, we selected a region with consistent signals in all 3 detection channels and where no sample movement occurred between all measurements, and the average signal over this region in each of the three detection channels was calculated using FIJI’s “plot z-axis profile” function and saving the result for each color consecutively.

To quantify the signal-to-background ratio as a function of the imaging depth, we analyzed the signal distribution in each *z*-plane image in a *z*-stack. The background (for each detection channel) was observed in parts of each image where no fluorescent molecules (that emitted in the corresponding channel) were present in the focal volume. In samples with a sufficiently sparse distribution of fluorescent structures (which was the case for our measurements), these regions are represented by the most abundant pixel value, and this value can be regarded as the background value. This would be equivalent to zero, or the global offset value, in a very thin sample or at the topmost slice in an image stack. To avoid division by zero when calculating the signal-to-background ratio, we chose the zero-value to be one less than the automatically detected background in the first (uppermost) slice in each image stack, i.e., the background was set to be 1 in the topmost slice. Several different strategies could be devised to obtain the signal value from the image. Depending on the nature of the image, the best strategy will be different. We found that deriving the signal value using a threshold in the histogram (e.g., the highest pixel value that occurred more often than the threshold in the histogram, or as the histogram value where more than the threshold fraction of pixels was brighter) allowed us to obtain signal values that consistently slowly varied between consecutive planes and gave plausible values for the signal-to-background contrast. The choice of the threshold value obviously directly influences the extracted signal-to-background ratio, and the same settings yield different results depending on what proportion of the image consists of bright pixels (pixels with signal), but it allows for an automated, unbiased determination of the signal level. We set the signal threshold conditions the same for each color in any image stack. The signal value for each slice is then the difference between the signal threshold location (pixel value) and the histogram maximum location (pixel value). Between measurements of different samples, we selected the signal threshold conditions such that the calculated contrasts for the first few (topmost) slices roughly matched between different measurements.

For displaying purposes, we applied low-pass Fourier filtering to the images to reduce pixel noise. The spatial cut-off frequency was chosen such that it was well above the frequency corresponding to the spatial resolution of the system, i.e., the resolution of the images was not reduced nor enhanced by the processing; only high-frequency pixel noise was removed. For visualization, the colors and contrast/brightness settings in the images recorded were chosen to show the structure of the fluorescence most clearly. Hence, the colors do not represent the true color of the light detected. In stacks, the brightness and contrast settings were adjusted such as to show the presence of the signal at the top layers, but also attempted to allow for as much visibility of signal variations at deeper layers. Thus, the ‘orange/red’ (570–640 nm) channel was displayed using the ‘red’ color scheme in FIJI, the ‘green’ (490–560 nm) channel using the ‘green’ color scheme, and the ‘blue’ (400–480 nm) channel using the ‘cyan’ color scheme.

## 3. Results

### 3.1. Two-Photon and 3P Excitation PSF Measurements

**Figure 5:**
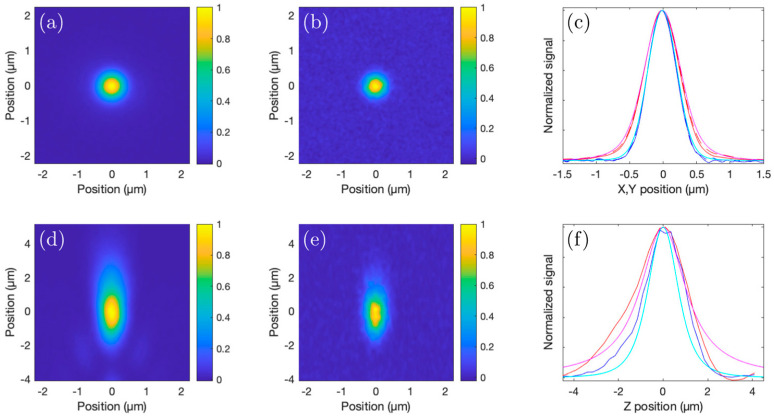
Three-dimensional measurement of the 2P and 3P PSF. (**a**,**b**) Projections of the 3D measurement along the *z*-axis of the orange (2P) and blue (3P) fluorescence, respectively, from a 500 nm bead. (**d**,**e**) Projections along the *y*-axis of the orange and blue fluorescence, respectively, of the same 500 nm bead, to visualize the *z*-profiles of the PSFs. (**c**) Linear lateral cross-sections of the 2P and 3P PSFs. The solid lines represent the *x* cross-sections and the dashed lines show the *y* cross-sections of the 3P (blue) and 2P (red) PSFs. The magenta and cyan lines show the lateral cross-sections of the calculated 2P and 3P PSFs, convoluted with a 500 nm sphere, respectively. (**f**) Linear axial cross-sections of the measured 2P (red line) and 3P (blue line) PSFs. The magenta and cyan lines show the axial cross-section of the calculated 2P and 3P PSF convoluted with a 500 nm sphere, respectively.

We experimentally estimated the three-dimensional 2P and 3P PSFs using a microscope slide with sub-micrometer-sized beads, each labeled with four different fluorophores (TetraSpeck, Invitrogen, Waltham, MA, USA). The blue (365/430 nm (single-photon excitation/emission wavelengths)) fluorophore was used to determine the 3P PSF, and the orange (560/580 nm) fluorophore was used to determine the 2P PSF. Stacks of *x*–*y* images with the orange and blue fluorescence recorded simultaneously were acquired from 500 nm diameter beads, with each acquired plane separated by 250 nm (see Figure 5a–d). The measured *x-*, *y-,* and *z*-projections agreed well with the calculated shape of the focus of a 1040 nm beam with a minimal beam waist (1/e^2^ radius) of 0.55 µm, taking into account the 500 nm size of the beads. Theoretically, when overfilling the back aperture of a 1.05 NA objective, the focal spot size (1/e^2^ radius, *w*_0_) of a 1040 nm (λ) beam would be about $w_{0}=0.41\lambda /NA\approx \;$0.4 µm. Our measured focus was larger, as our beam size did not overfill the back aperture of the objective (owing to the 5 mm aperture of the scanning mirrors and four-fold magnification offered by the scan lens–tube lens pair). Figure 5d,e show the PSFs of the focused beam along the beam propagation direction for 3P and 2P fluorescence. The lateral 1/e^2^ radius of the 3P PSF is 0.32 µm, and for the 2P PSF, it is 0.39 µm.

### 3.2. Simulations

To illustrate the improvement in the signal-to-background ratio that can be obtained with 3P excitation, we simulated 2P and 3P signals generated in samples with different scattering lengths, with the focusing conditions set to match the experimentally measured 2P and 3P PSF. Other works, e.g., [12,30], have presented similar analyses; however, these have used a different approach to the approach presented below.

When propagating in a medium with a scattering length, *l_e_* (defined as the characteristic length over which a fraction of 1/e photons is not scattered), and refractive index, *n*, the intensity (*I*) of a focused beam with a minimal 1/e^2^ waist (*w*_0_) at *z* = 0 and wavelength (λ), normalized to the intensity in the focus, is as follows:

(1) $$I\left(x,y,z\right)=e^{-\left(2x^{2}+2y^{2}\right)/w{\left(z\right)}^{2}}\frac{w_{0}^{2}}{w{\left(z\right)}^{2}}e^{\frac{z}{l_{e}}}$$

with the beam waist, *w(z),* given as

(2) $$w\left(z\right)=w_{0}\sqrt{1+\frac{z}{n\;z_{R}}}$$

and the Rayleigh length, *z_R_*, given as

(3) $$z_{R}=\pi w_{0}^{2}/\lambda$$

Note that as we define the focal plane as *z* = 0, as well as a positive *z* above the focus (the beam propagates in the direction of negative *z*), the final factor in Equation (1), denoting the loss of ballistic photons towards the focus, increases with positive *z*.

The signal, *S*(*x,y,z*), generated at each point in a volume with a homogenous distribution of fluorophores is proportional to *I^m^*, with *m* = 2 for 2P excited fluorescence and *m* = 3 for 3P excited fluorescence. We calculate the signal-to-background ratio as a function of the imaging depth (*z*) by integrating the signal, *S*(*x,y,z*), generated in the focal volume (within ±5.8 µm, corresponding to about ±3.5 times the Rayleigh length in the medium) and dividing this by the integrated signal (*S*) above the focal volume (starting at 5.8 µm) up to a height (*z*) (the geometric focus is located at height 0) when taking an exponential loss in power into account for the beam propagation. Theoretical estimations of signal-to-background in other works [12,30] have excluded a larger distance from the focal plane when calculating the background, which tends to result in underestimating the background; this is valid for long scattering lengths but breaks down for more high-scattering samples.

**Figure 6:**
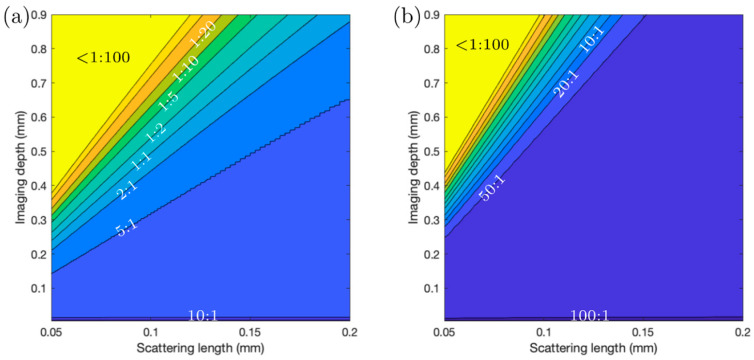
Simulated signal-to-background ratio vs. scattering length and imaging depth for (**a**) 2P excited fluorescence and (**b**) 3P fluorescence. Contour lines (iso-values for signal-to-background ratio) and color coding are the same for both panels. Contour lines are labeled where possible. Beyond a signal-to-background ratio of 1:20, contour lines are drawn at 1:50 and 1:100. Obviously, those last contours represent situations where image quality is so gravely compromised that imaging is practically impossible.

An additional factor leading to overestimating the signal-to-background ratio is signal absorption, which can be especially significant when imaging deeper into the sample. Our simulations allow us to account for this by attenuating the fluorescence as a function of depth with an absorption length of ~0.5 mm. To account for the fact that there will be space without fluorescent molecules between spaces with fluorescent molecules (e.g., water or growth medium between the bacteria), the signal in the focus is multiplied by a factor of 10. That is, we assume that bacteria occupy 10% of the volume of the sample, and from 90% of the volume, no signal is generated. The 10% of the volume occupied by *Streptomyces* bacteria is estimated from the acquired images by finding the fraction of pixels in an image higher than a certain threshold that captures all bacteria but not including the background. For comparison, in mouse brain imaging, only about 1% of the tissue is labeled, and 10 times less background will be generated compared to our *Streptomyces* samples. Figure 6 shows comparisons of signal generation vs. depth and scattering length for 2P and 3P imaging. The loss in the signal-to-background ratio at increasing depths for 2P imaging can be observed to be worse than for 3P imaging.

Just as the scattering in our bacterial community sample is linked to the density of the sample, the average number of fluorescent molecules per unit volume also increases with increasing density, but the number of fluorescent molecules in the focal volume for a ‘bright’ pixel (which is determined based on the number of fluorescent molecules inside a single bacterium) remains constant, given that the bright pixel volume is smaller than a single bacterial cell. Thus, the signal-to-background ratio will also scale inversely linearly with the sample (labeling) density, as was pointed out as well in other treatments of the signal-to-background ratios in 2P and 3P imaging [12,30]. Incorporating sample sparsity as an additional parameter into our simulations complicates the visualization of the results, and as it effectively just constitutes a linear scaling, we only focus on the scattering length in our simulations. While a significant improvement (roughly doubling the imaging depth) in relatively sparse samples such as the mouse brain has been observed when comparing 2P and 3P imaging, the improvement may be far more impressive when imaging samples with a higher signal density and higher scattering, such as bacterial communities, biofilms, and plant tissues.

### 3.3. Characterization of the Nonlinear Nature of the Fluorescence

**Figure 7:**
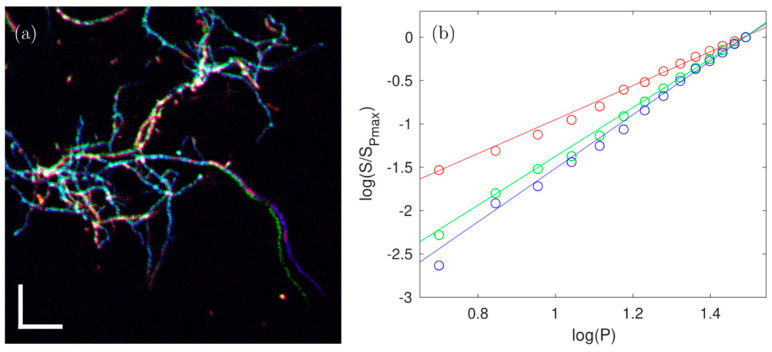
Characterization of the nonlinearity of the fluorescence in different detection spectral windows. Panel (**a**) shows a composite image (76 µm × 76 µm) of the measurements in all 3 detection channels at the highest laser power shown in panel (**b**). Scale bars: 10 µm. A region where the no sample movement was detected between all measurements was chosen to extract the signal vs. background. Panel (**b**) shows the power dependence of the fluorescence signal in the red (570–640 nm), green (490–560 nm), and blue (400–480 nm) detection channels.

For imaging purposes, signals from *S. aizunensis* were collected in three different spectral windows, as described above. It must be noted that no exogenous labeling was applied; thus, the signal relied only on the natural fluorescence from the sample. The three-color image of a ~30 µm thin sample with an ensemble of signals from *Streptomyces* filaments is shown in Figure 7a. This sample was used to obtain a power dependence of the fluorescence signal in each of the three spectral windows, in order to reveal the (dominant) nature of the excitation of the fluorescence in each channel. A logarithmic plot of the signal in each of the spectral windows (divided by the signal obtained at the highest power used) vs. the excitation power for each of the detection channels is shown in Figure 7b.

The data show that the signal (*S*) detected in the ‘blue’ channel increased with the third power of the excitation power, *P* (*S_blue_ = P*^3.09^), and thus is exclusively due to 3P excitation. The signal detected in the red channel increased with the square of the excitation power (*S_red_* = *P*^1.96^) and can be attributed to be (almost) exclusively 2P excitation. Finally, the signal detected in the green channel increased with a power dependence of 2.81 (*S_green_* = *P*^2.81^), indicating it has contributions from both 2P and 3P excitation. This may indicate that more than one fluorophore emits in the 490–560 nm window, but it is also possible that one fluorophore can be excited with both 2P and 3P excitation, as was demonstrated by Hontani et al. [28].

### 3.4. Comparison of 2P and 3P Imaging Performance

**Figure 8:**
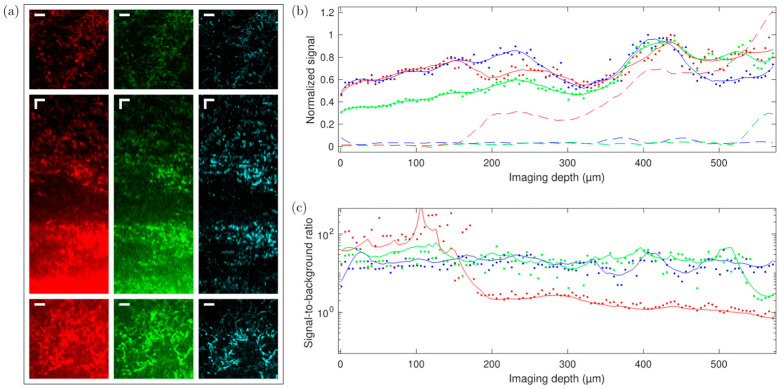
Measurement of 2P and 3P fluorescence vs. depth (76 µm × 76 µm × 570 µm) in a Streptomyces sample. (**a**) Red (570–640 nm), green (490–560 nm), and blue (400–480 nm) fluorescence channels (top row—*xy* slice 90 µm below top surface; middle row—central *xz* cut; bottom row—*xy* slice 390 µm below top surface; horizontal (*xy*) scale bar: 10 µm; vertical (*z*) scale bar: 30 µm). (**b**) Signal minus background vs. depth normalized to maximum value (solid dots and solid lines) and, accordingly, scaled background vs. depth (dashed lines) for the red, green, and blue channels. (**c**) Signal-to-background ratio vs. depth for the red, green, and blue detection channels.

**Figure 9:**
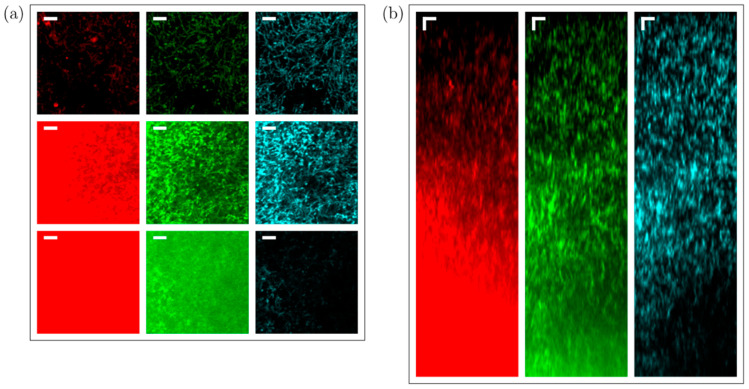
Measurement of 2P and 3P fluorescence vs. depth (76 µm × 76 µm × 270 µm) in a dense *Streptomyces* bacterial community sample. (**a**) Red (570–640 nm), green (490–560 nm), and blue (400–480 nm) fluorescence channels (Top row—*xy* slice 10 µm below top surface. Middle row—*xz* slide 160 µm below top surface. Bottom row—*xy* slice 270 µm below top surface. Scale bar: 10 µm.). (**b**) Center *xz* cut; scale bar: 10 µm.

**Figure 10:**
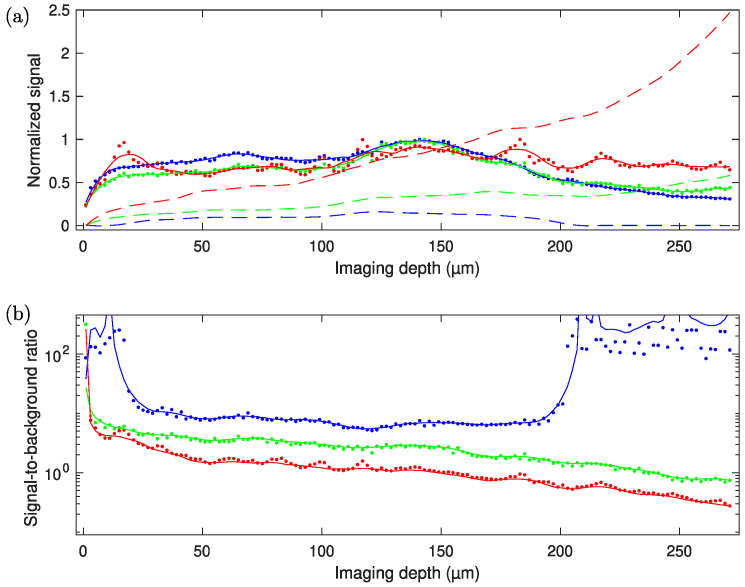
(**a**) Signal minus background vs. depth corresponding to the stack shown in Figure 7, normalized to maximum value (close to 150 µm depth; solid dots and lines) and, accordingly, scaled background vs. depth (dashed lines) for the red, green, and blue channels. (**b**) Signal-to-background ratio vs. depth for the red, green, and blue detection channels.

To demonstrate the superior contrast provided by 3P imaging deep in scattering samples, we acquired image stacks from columns filled with mycelia of *S. aizunensis*. By varying the water content in the sample, the density (and consequently, the scattering attenuation length) of the bacterial filaments can be changed. Figure 8, Figure 9 and Figure 10 show the results from two samples with significantly different densities. The attenuation (scattering) length of a sample can be determined by comparing the power needed at different depths for similar image brightness to an exponential law. The denser sample has an estimated attenuation length of ~80 µm, and the more diluted sample has a corresponding attenuation length of ~170 µm. Again, no exogenous fluorophore was added, meaning that we purely relied on the autofluorescence of the sample. The samples were imaged down to a depth where the laser power needed to achieve the same 3P signal, as just below the surface required 100% of the available power (which resulted in ~60 nJ—250 mW—on the sample surface). Especially in the ‘orange/red’ channel, the increased background causes the images to appear saturated, even though the signal and background can be distinguished readily in the data. Images at the top and bottom planes cannot be displayed with the same brightness/contrast settings, yet at the same time, show an equal level of detail.

Figure 8 shows the imaging results from the less-dense sample, where we imaged down to a depth of 570 µm below the surface of the sample. For the ‘orange/red’ channel, the signal equals the background at around 550 µm depth, while for the ‘blue’ channel, the background stays almost constant through the length of the stack that was measured (see Figure 8b). For the ‘green’ channel, where both 2P and 3P excitation processes are associated with fluorescence generation, the background stays almost constant up to a depth of 500 µm, where it starts increasing. The scattering length in this sample is estimated to be 170 µm. The signal-to-background ratio of the blue, 3P excited fluorescence does not noticeably degrade throughout the entire column, and remains better than 10:1, while the green (combined 2P and 3P) and orange/red (2P excited) fluorescence signals degrade from better than 20:1 to about 2:1 and 1:1.5, respectively, at the last plane imaged. It may be noted that the green fluorescence signal appears to gradually increase with depth (the signal at the deepest planes imaged is about double the signal at the topmost planes), but the blue and red fluorescence signals are roughly equal at the topmost and deepest planes. While we do not have a definite explanation as to why the signal in the green channel appears to increase with depth relative to the blue and red signals, it may simply be due to a natural variability in the concentration of the fluorophore(s) responsible for green fluorescence in the streptomyces, independent of the variability in the concentration of the red and blue fluorophores. As the orange/red fluorescence was roughly the same in both the image stacks taken with the ‘blue’ and ‘green’ filters, we do not expect that photobleaching of the green fluorophore was caused during the measurement with the ‘blue’ filter, although it cannot be ruled out. As we performed measurements in different regions in the sample while alternating whether the green or blue fluorescence was acquired first, with a reproducible signal-to-background dependence of all three wavelength ranges, we can conclude that out-of-focus photobleaching remains insignificant, and is therefore unlikely to have caused the green fluorescence to be relatively weaker at the top of the sample in the measurement shown in Figure 8. All three fluorescence colors show more signal at around the 400 µm depth, which can also be attributed to the natural variability in fluorophore concentrations. The relative increase in the green-channel signal with depth, however, causes the signal-to-background ratio to deteriorate slower in the green channel, compared to a situation where it would be similar throughout the entire depth of the sample. Figure S1 shows an image stack of a similar sample obtained on a confocal microscope. Figure S2 shows brightfield and widefield epifluorescence images obtained from a sample similar to the samples used to obtain Figure 8, Figure 9 and Figure 10.

Figure 9 and Figure 10 show the imaging results from the higher-density sample of *S. aizunensis*. In this sample, we could image from the surface down to a depth of 270 µm with the available laser power due to a reduction in the scattering length to an estimated 80 µm. In these images, the signal-to-background ratio in the blue, 3P excited fluorescence is about 100:1 at the top, degrades to values around 10:1 between 10 µm and 200 µm below the sample surface, and again becomes better than 20:1 below the 200 µm depth. The ‘green’ (mixed 2P and 3P) and ‘orange/red’ (2P) fluorescence signals degrade from better than 100:1 at the top to almost 1:1.5 and 1:3.5, respectively, at the 270 µm depth. The measurements in this sample also corroborate the assumption that out-of-focus photobleaching is not likely to be the cause of the lower ‘green’ signal at the upper ~150 µm in Figure 8, as here, the ‘green’ and ‘orange/red’ signals follow roughly the same trend in the upper ~150 µm. In fact, in this sample, the ‘orange/red’ signal appears relatively stronger towards the lower imaged layers. The results obtained with the sample with a shorter attenuation length show that with a limited excitation power, the signal-to-background ratio at the maximum achievable depth for that power deteriorates with decreasing attenuation length.

## 4. Discussion and Conclusions

Bacterial communities/biofilms serve as examples of high-scattering biological samples, as live bacteria have a variety of biomolecules that show characteristic excitation and emission spectra, which can be used for identification and characterization purposes [1]. Two-photon LSM has been successfully applied for the high-resolution deep imaging of dense oral biofilms [16]; bacteria differentiation and high-resolution imaging within deep in vitro biofilms [17]; to visualize bacteria and extracellular secretions within marine stromatolites [15]; and for the assessment of fluorochromes for 2P excitation applications in biofilms [18]. Two-photon excitation, in combination with fluorescence correlation, was applied to study diffusion inside microbial biofilms [19], and in combination with fluorescence lifetime imaging, to study the activity of bacteria in stained lotic biofilms [13], as well as the spatial distribution of zinc in microbial biofilms [20]. No studies on 3P LSM for the in vivo imaging of dense microbial samples have been reported. Non-destructive methods, such as isotope probing coupled to confocal or Raman microscopy, have been used to study the spatial arrangement and metabolic activity of bacterial biofilms [31]. Another recent study used stimulated Raman scattering (SRS) microscopy to image thin sections of *Pseudomonas aeruginosa* biofilms to visualize phenazine distribution, a marker for biofilm formation during infection [32]. This non-label-free method of visualizing the spatial distribution of methylated phenazine, that supports biofilm activity at depth, allowed them to characterize strains that do not methylate phenazine and hence are unable to form thick biofilms [33]. Another study using various bacterial species used the autofluorescence of anthracyclines, entrapped in extra-cellular vesicles, to demonstrate membrane fusion into the target organism, B. subtilis [34]. In this study, we develop a new method of 2P and 3P label-free imaging of intact *Streptomyces* bacterial communities exhibiting intrinsic fluorescence and demonstrate how the method allowed us to image several hundred µm deep into the samples.

Most sources used for 3P microscopy, especially those operating around wavelengths of 1.3 µm and 1.7 µm, are based on frequency conversion schemes like soliton frequency shifting and optical parametric amplification [10,35,36,37,38,39,40,41,42], as little to no laser materials suitable for direct ultrafast pulse generation at these wavelengths are available. Due to the relatively low 3P absorption cross-sections as compared to the 2P absorption cross-sections, 3P excitation requires a higher pulse energy to achieve sufficient excitation efficiency. The 3P excitation of fluorophores with (single-photon) absorption spectra in the deep blue/UV range requires shorter-wavelength (<1200 nm) excitation sources, with sufficiently high peak powers.

The high peak power pulses combined with the operation at the 1040 nm wavelength of the Yb amplifier allowed for the simultaneous label-free 3P and 2P excitation of the endogenous fluorescence in the *Streptomyces* samples. The 3P and 2P excited fluorescence signals were stable, and signal degradation was drastically reduced compared to confocal microscopy with single-photon excitation. Fluorescence was recorded in three different spectral windows (400–480 nm, 490–560 nm, and 570–640 nm). The signal generation stability and low signal degradation observed with the 1040 nm multiphoton excitation light is very advantageous for the fluorescent characterization of *Streptomyces* bacterial communities, especially since quantitative analysis is very challenging due the presence of strong photobleaching and sample damage with single-photon excitation (confocal microscopy) approaches. The 1040 nm Yb-fiber chirped pulse amplifier system is especially attractive for the 3P excitation of fluorophores emitting in the blue region, since no additional frequency conversion schemes are required and the energy delivered by conventional, high-repetition-rate, tunable Ti:sapphire oscillators tuned to operate around 1040 nm is limited to just a few nJ. Even though our laser uses an all-fiber amplification scheme, the well-matched compression and stretching scheme allowed to produce clean pulses with well-compensated third-order dispersion, which helps to reduce unwanted heating effects in the samples; this is especially important for high-energy pulses for 3P excitation delivered at a MHz repetition rate and for imaging deep into scattering samples.

Light scattering and absorption limit the maximal depth in a sample at which it is possible to acquire images with a certain (user-defined) contrast. The reason behind this is explained as follows: As scattering and absorption reduce the peak intensity in the focus, to maintain a ‘constant’ signal strength generated from the focus when increasing the depth of the focus below the sample surface, the average power on the sample surface needs to be increased accordingly (to maintain a constant peak intensity in the focus). While the case of single-photon (confocal imaging) absorption should not be ignored, it can be considered insignificant in most cases involving 2P and 3P imaging (especially below 1400 nm). The loss of ballistic photons due to scattering can eventually lead to a situation in which the fluorescence (background) generated through multiphoton excitation outside the focus (mostly above) is comparable to, or even more than, the amount of fluorescence (signal) generated in the focus. Hence, for 2P and 3P imaging, the maximal imaging depth is directly linked to the scattering length of the sample. The population density (which could be expressed in terms of the number of cells per unit volume or the fraction of the volume occupied by bacterial cells) of a bacterial community influences the scattering length, and we observe that the loss in image contrast indeed is crucially influenced by scattering when we image into bacterial communities with different population densities.

**Figure 11:**
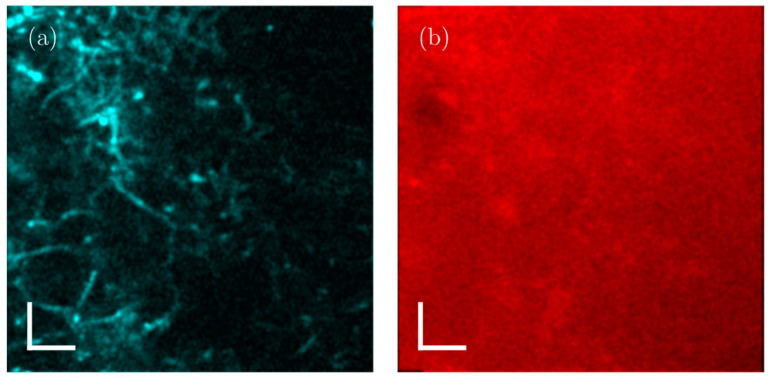
Images (76 µm × 76 µm) obtained at 780 µm below the sample surface in a medium-dense sample (with 120 µm effective attenuation length), i.e., at 6.5 scattering lengths below the surface; (**a**) 3P excited fluorescence signal detected in the 400–480 nm spectral window; (**b**) 2P excited fluorescence signal detected in the 570–640 nm spectral window. *Streptomyces* filaments are clearly recognized in the 3P image but cannot be resolved in the 2P image due to the overwhelming background. Scale bars: 10 µm.

It is important to note that the fraction of the image area occupied by fluorescent structures in the *Streptomyces* samples is about 10%, as compared to about only 1% that is covered by labeled neurons in the cortex of mouse brains, as presented in [29]. This causes background fluorescence to be 10 times stronger in *Streptomyces* samples compared to mouse brain imaging. Our observations indicate that the depth limit is not linearly dependent on the scattering length (or the density of the sample). Experimentally, we explored the 3P imaging depth at the maximum available output power from our laser system for a 120 µm scattering length (medium-dense sample). An imaging depth of ~780 µm could be achieved (see Figure 11), while our simulations show that for the 3P excited fluorescence, the signal-to-background ratio would be 1:1 at ~800 µm. The brightness and contrast settings for the images in Figure 11 are set to allow for an optimal visualization of the signals, causing the signal-to-background to be slightly enhanced in the images. The lack of detail in the 2P fluorescence signal in Figure 11b, however, is the direct effect of the strongly degraded signal-to-background ratio at 6.5 scattering lengths below the surface. Inhomogeneities in the sample cause slowly varying inhomogeneities in the background that are responsible for the observed spatial variations in the 2P image (Figure 11b). The laser power is limited by the available pump power from the diode lasers used to pump the last amplification stage, but the output power can be still increased straightforwardly by increasing the available pump power for the last amplification stage, without running into detrimental nonlinearities that can degrade pulse compressibility.

Since the fluorophore concentration is not determined by exogenous labeling but by the natural growth of the sample, as well as the sample heterogeneity, the detected signal strength is not just determined by the imaging depth of the sample. No monotonic decrease in the signal is observed, but for the sample with an estimated 170 µm scattering length (low bacterial density), the background signal difference between the blue (3P excitation) and red channels (2P excitation) starts to be significant at a depth of ~200 µm, and for the sample with an 80 µm scattering length (high bacterial density), the background signal difference starts to be significant at a depth less than 100 µm below the surface.

Our work can be compared and contrasted with prior efforts of combined 2P and 3P excitation that has been applied for the simultaneous imaging of neural activity at separated axial planes. This was achieved through the temporal multiplexing of the 2P (920 nm) and 3P (1320 nm) excitation source [39,43], as well as remote focusing [44] to achieve imaging in different axial planes. In this paper, we have demonstrated simultaneous deep, label-free 2P and 3P imaging in *Streptomyces* communities using a single Yb-fiber laser source with a central wavelength of 1040 nm. The simultaneous 2P and 3P excitation of endogenous fluorophores in *Streptomyces* bacteria was enabled through the single-source excitation and the high peak power delivered by the presented Yb-fiber laser.

In summary, we present a multicolor imaging platform that can be applied to investigate structure, spatial patterning, the dynamic trafficking of endogenous metabolites, and for the quantitative characterization of fluorescence at depths not achievable with single-photon excitation approaches. In this work, we exploited intrinsic fluorescence in the *Streptomyces* samples and described a multicolor imaging tool that has the potential to be of relevance for imaging approaches of other microbial communities. The combined 2P and 3P approach is not limited to the use of endogenous fluorophores, but can also be readily used with exogenous fluorophores.

## Data Availability

Data underlying the results presented in this paper are not publicly available at this time but may be obtained from the authors upon reasonable request.

## Supplementary Materials

The following supporting information can be downloaded at https://www.mdpi.com/article/10.3390/s24020667/s1: Figure S1: Confocal imaging data, Figure S2: Brightfield and widefield epi-fluorescence image of a mm thick sample.
