# Supplementary material for: Simultaneous Two- and Three-Photon Deep Imaging of Autofluorescence in Bacterial Communities
Source: Sensors (Basel). 2024 Jan 20;24(2):667. doi: 10.3390/s24020667 (PMC10819415; doi:10.3390/s24020667)
Supplement: Supplementary file 1 [file sensors-24-00667-s001.zip › sensors-2812435-supplementary.pdf]

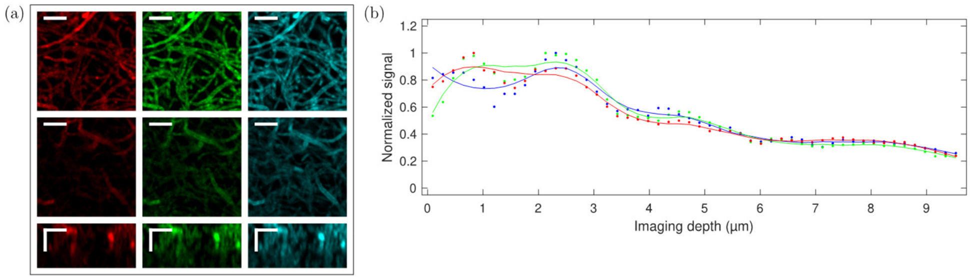

Figure S1. Imaging stack (21.44×21.44×9.62 μm) of *Streptomyces* bacterial community measured with a single photon confocal microscope. (a) Slices at ~0.5 μm (top row), and 9.0 μm (second row) below the sample surface and a center *xz*-cut (bottom row). Scale bars 5 μm. (b) Normalized signal (dots) extracted from the imaging stack. The solid lines are a Savitzky-Golay smoothing of the dots, red, green and blue correspond to red (561/605 nm excitation/emission), green (488/525 nm excitation/emission) and blue (405/450 nm excitation/emission) detection modes, respectively. The sample was about 30 μm thick, however the severely degrading image quality limited acquisition to just 9-10 μm.

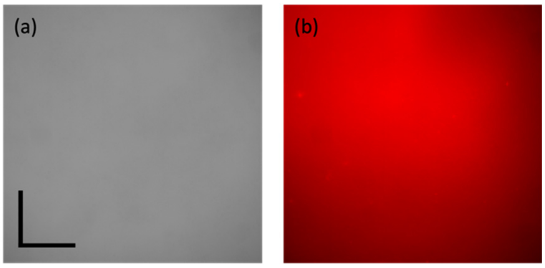

Figure S2. Images taken in brightfield (a) / widefield (b) mode from a 1 mm thick *Streptomyces* bacterial community sample, similar to the samples described in Section 3.4. The strong scattering from this sample causes an overwhelming blur even when the focal plane is directly below the cover slip. The widefield epifluorescence image (b) is taken using an RFP excitation/emission filter set. Scale bars 100 μm.
